# Successful treatment of nail lichen planus with tofacitinib: a case report and review of the literature

**DOI:** 10.3389/fmed.2023.1301123

**Published:** 2023-11-16

**Authors:** Jundong Huang, Wei Shi

**Affiliations:** Hu Nan Key Laboratory of Aging Biology, Department of Dermatology, Xiangya Hospital, Central South University, Changsha, China

**Keywords:** nail lichen planus, tofacitinib, case report, review, JAK inhibitor

## Abstract

Nail lichen planus (NLP) is a chronic inflammatory disease of unknown etiology and has been recognized as a nail potentially critical disorder, which can be severe and rapidly worsen with irreversible scarring. Currently, the treatment options are limited based on disease progression. High-potency topical or intralesional corticosteroids are commonly considered first-line therapeutic options; however, these therapies are unsuitable for all patients with NLP, especially those with extensive lesions. As a potential therapeutic target for inflammatory skin diseases, Janus kinase (JAK) inhibitors can suppress both type-1 and type-2 cytokines, thereby reducing the immune response and resultant inflammation. Recent studies have suggested benefit in cutaneous lichen planus and lichen planopilaris with oral JAK inhibitors. Here, we report a case of severe NLP that exhibited a favorable response to tofacitinib treatment. A 41-year-old woman presented to our clinic with a 2-year history of nail dystrophy of all fingers of both hands. The NLP was finally confirmed by histopathology and the above clinical features. After the informed consent signature, tofacitinib monotherapy, 5 mg twice a day, was then begun, and after 6 months, the appearance of her nails had a significant improvement.

## Introduction

Nail lichen planus (NLP) is a rare inflammatory nail disorder, which usually emerges in adulthood, and is characterized by predominant nail matrix destruction that can affect one or multiple nails/toenails (1). The management of NLP has always been challenging because of high rates of therapeutic failures and limited treatment options available, especially at the stage of dorsal pterygium (1).

As a potential therapeutic target for inflammatory skin diseases, Janus kinase (JAK) inhibitors can suppress both type-1 and type-2 cytokines, thereby reducing the immune response and resultant inflammation (2). Here, we report a case of severe NLP that exhibited a favorable response to tofacitinib treatment.

## Case presentation

**Figure 1:**
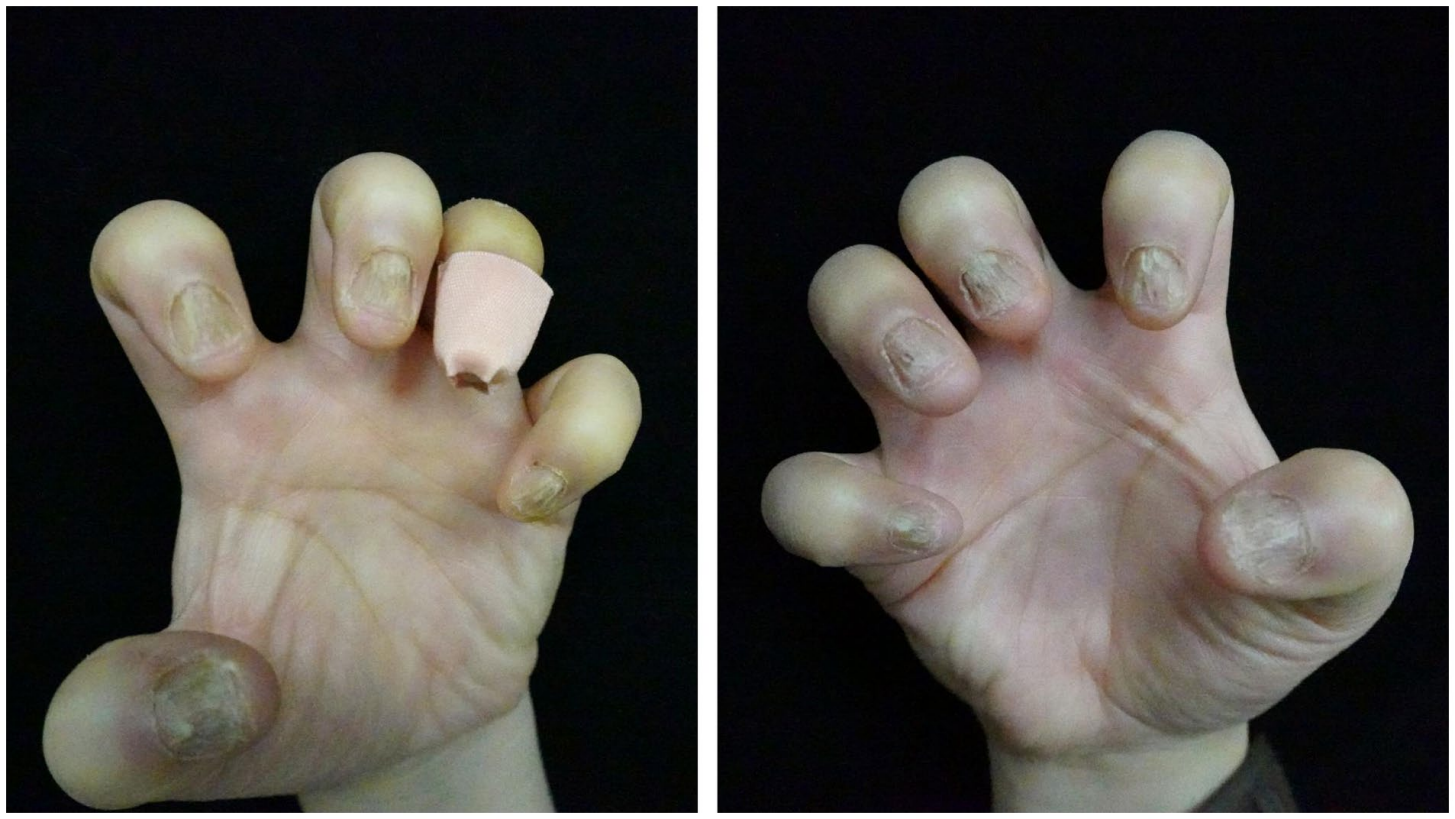
Clinical manifestation of nails of both hands before tofacitinib treatment.

A 41-year-old woman presented to our clinic with a 2-year history of nail dystrophy of all fingers of both hands. With seriously impacted qualities of life, she had a much more urgent demand for effective therapies. Onychomycosis was ruled out by direct microscopy and the history of failure of anti-fungal therapy. Physical examination demonstrated a series of typical NLP symptoms of longitudinal ridges and grooves, distal splitting, and trachyonychia, while nail bed and nail fold were generally normal (Figure 1). The patient was otherwise healthy and no other skin lesions were noted. Histological examination of nail biopsies showed band like lymphocytic infiltrate of nail matrix and focal liquefaction of the basal layer. Her NLP was finally confirmed by histopathology and the above clinical features.

**Figure 2:**
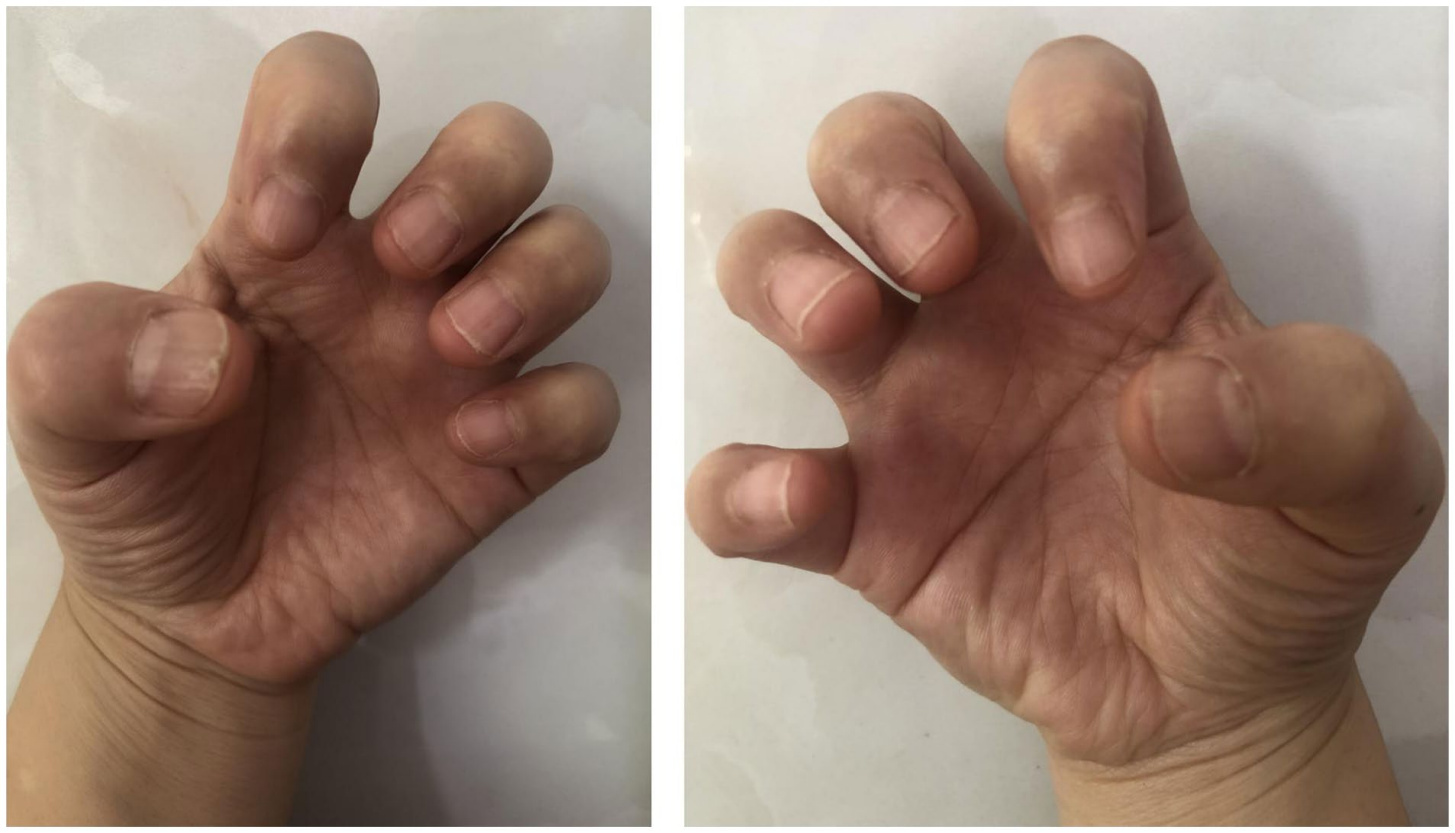
Clinical manifestation of nails of both hands after 6 months of treatment with tofacitinib, 5 mg twice a day.

The patient was initially treated with the topical application of high-potency corticosteroids and tacrolimus 0.1% ointment under occlusion using plastic film for 3 months, however, no significant improvement was seen. Due to the high number of nail involvement and painful side effects of the injection process, intralesional triamcinolone acetonide was not considered. We were also hesitant to initiate systemic treatment with oral retinoids or immunosuppressive agents, which is associated with increased risks and high treatment failure rates (3). Routine laboratory testing did not identify any abnormalities in our patient and screening for hepatitis B virus and latent tuberculosis infection was also negative. Tofacitinib monotherapy, 5 mg twice a day, was then begun, and after 6 months, the appearance of her nails had a significant improvement (Figure 2). During follow up, no treatment related adverse reactions were reported. As of this writing, the patient continues to receive tofacitinib and is doing well.

## Discussion

Nail lichen planus (NLP) is a chronic inflammatory disease with either isolated nail involvement or concurrently skin and mucosal involvement. It also has been recognized as a nail potentially critical disorder, which can be severe and rapidly worsen with irreversible scarring (4). High-potency topical or intralesional corticosteroids are commonly considered first-line therapeutic options; however, these therapies are not suitable for all patients with NLP, especially those with extensive lesions (5).

Although multifactorial in etiology, the mechanisms leading to lichen planus (LP) are not fully understood but likely involve a combination of genetic predisposition and environmental triggers, eventually leading to the auto-immune attack of the basal keratinocytes mediated by CD8+ CXCR3+ cytotoxic T cells and plasmacytoid dendritic cells (6). There is growing evidence showing that LP is a Th1-driven disease and mediates key cytokines in its pathogenesis through the JAK–STAT pathway (7). These finding suggest that tofacitinib might also have therapeutic benefits in NLP. Moreover, recent studies have suggested benefit in cutaneous lichen planus and lichen planopilaris with oral JAK inhibitors (7, 8). According to the above discussion, after clinical evaluation, treatment with tofacitinib was initiated with off-label use in this case.

Tofacitinib is a representative pan-JAK inhibitor and strongly blocks JAK1/3 but weakly inhibits JAK2 (9). It was first approved in 2012 by the US Food and Drug Administration (FDA) for the treatment of moderate to severe rheumatoid arthritis and has since expanded the indications include psoriasis, psoriatic arthritis and ulcerative colitis (9). However, as a non-selective first-generation JAK inhibitor, the clinical success of tofacitinib is limited by the increased adverse side effects associated with its broad inhibitory activity. In a study of seven-year safety databases based on the post-marketing surveillance data, a statistically significant elevation in the risk for pulmonary embolism and overall mortality were noted with the 10-mg dose of tofacitinib (10). Moreover, another randomized, noninferiority, safety end-point trial showed that the incidences of major adverse cardiovascular events and cancer were higher with the combined tofacitinib doses (3.4 and 4.2%, respectively) than with a TNF inhibitor (2.5 and 2.9%, respectively) (11). As a result, the FDA added a black-box warning and modified the labeling for all currently approved JAK inhibitors across all indications. It must be emphasized that all the above mentioned findings were based on patients with rheumatoid arthritis, which can only partially be generalized to patients with skin disorders. Identifying potential risk factors (include age>65, obesity, smoking, cardiovascular disease, coagulation disorder, or history of thromboembolism or malignancy) in an average individual may help in making tailored decisions in clinical practice (12).

The patient ultimately chose tofacitinib for the following reasons: (1) extensive nail damage with topical or intralesional corticosteroids is unsuitable because of the pain and inconvenience of that procedure, patient compliance is often poor; (2) systemic corticosteroids (0.5–1 mg/kg/d) are the most commonly used treatment options in clinical practice for severe LP, which may lead to a wide array of adverse events (3). Furthermore, based on our clinical experience, we found that females and elders were more vulnerable to the adverse effects of corticosteroids. The patient in our case refused glucocorticoid therapy because of obesity and menstrual disorders that might be caused by the inability to receive glucocorticoid therapy; (3) several reviews on the overall safety of JAK inhibitors have been published recently, suggesting that JAKi has good security with a low risk of venous thromboembolism, cardiac events, and cancer (13–17). As such, follow-up according to guidelines greatly ensured patient safety.

To date, there have been only two case reports about JAK inhibitors for the treatment of NLP (18, 19). In one case, an unexpected improvement in the nails was observed after the use of tofacitinib due to concomitant rheumatoid arthritis (19). Even more to the point, a successful pilot study indicated that tofacitinib yielded significant remission of nail lesions in patients with Synovitis-Acne-Pustulosis-Hyperostosis-Osteitis (SAPHO) syndrome (20). Given that permanent nail loss may occur without prompt and effective treatment, we propose that use of the JAK inhibitor tofacitinib results in rapid resolution while avoiding the long-term side-effects of other topical treatments, such as corticosteroids. More studies are required to investigate the efficacy and remittive effect of tofacitinib for the treatment of NLP.

In order to fully describe the anecdotal reports of the successful treatment of nail lichen planus, a comprehensive literature search of PubMed was performed on October 29, 2023. Search terms included: “nail lichen planus,” “nail and lichen planus,” “treatment,” and “therapeutic.” In addition, articles including the wording “case report” or “case series” were added. All the sourced articles were full-text reviewed to ensure that the contents were relevant to the study. The literature search in PubMed yielded 155 articles. After the screening process, a total of 8 articles were labeled as eligible. Patient characteristics and treatment and response data are shown in Table 1. In summary, few cases of successful treatment of NLP have been reported. Low-quality research may lead to poor information for evidence-based decision-making.

**Table 1:** The anecdotal treatment-related case reports.

Study number	Author	Year	Sex (M/F)	Age, years, median/(range)	Disease duration, median (range)	Affected nails, n, median (range)	Patient with extra-nail involvement, n	Treatment	Treatment duration, median (range)	Response to treatment*	Patient-report Relapse, n
1	Duplaine and Cogrel (21)	2023	4 (M)/1 (F)	58 (51–70)	9 (1–11) years	20 (12–20)	Yes (2/5)	Mycophenolate mofetil (2–3 g/day)	15 (4–96) months	Mild improvement (1/5), moderate improvement (2/5), clinical cure (2/5)	Yes (2/5)
2	Milani and Adamo (22)	2022	6 (M)/4 (F)	38	NA	4 (1–10)	NA	A nail lacquer containing urea 20% and keratinase and hydroxipinacolone retinoate 0.1% (once a day)	12 weeks	At baseline, the NLPSS was 20.8 ± 3. After 12 weeks, the NLPSS showed a significant reduction to 4 ± 8.8	NA
3	Rehman et al. (23)	2021	4 (M)/10 (F)	(14–55)	6.5 (4–18) months	NA	Yes (9/14)	Combination of subcutaneous triamcinolone (every 2 weeks) and platelet-rich plasma (every 4 weeks) vs. subcutaneous triamcinolone only	24 weeks	In finger nails, good response was seen in 13 (50%) nails, moderate response in nine (34.6%) nails, and poor response in four (15%) nails in the combined therapy group; in toe nails, good response was seen in 12 (48%) nails, moderate response in four (16%) nails, and poor response in nine (36%) nails in the combined therapy group	NA
4	da Veiga et al. (24)	2020	1 (F)	52	12 years	10	No	Localized topical PUVA (three times a week)	1 year	The thumb plates had improved considerably	NA
5	Florian et al. (25)	2014	1 (F)	39	2 years	20	No	Cyclosporine 3 mg/kg (100 mg twice a day)	1 year	Complete remission	NA
6	Ujiie et al. (26)	2010	4 (M)/1 (F)	(11–58)	24 (4–84) months	NA	Yes (1/5)	0.1% topical tacrolimus (twice a day)	(15–71) months	All of the patients showed marked improvement	No
7	Irla et al. (27)	2010	1 (F)	53	5 years	NA	No	Etanercept (25 mg) subcutaneous injection twice weekly for the first 6 months and 50 mg subcutaneous injection once weekly thereafter	9 months	A marked improvement of her toe nail lesions	NA
8	Mostafa (28)	1989	1 (F)	40	4 years	20	Yes	Chloroquine phosphate (250 mg twice daily)	30 weeks	Complete remission	NA

*There is no unified methodology to evaluate the effectiveness. As such, efficacy was described according to the results of the included studies. NLPSS, nail lichen planus severity score; NA, not available; M, male; F, female.

In conclusion, NLP is a chronic inflammatory disorder with significant cosmetic and functional impact. The treatment options are limited based on disease progression. This case highlights the potential of the tofacitinib to improve this condition, and improve patient quality of life. More studies and observations are needed to fully appreciate the potential of tofacitinib for NLP.

## Data availability statement

The raw data supporting the conclusions of this article will be made available by the authors, without undue reservation.

## Ethics statement

Written informed consent was obtained from the individual(s) for the publication of any potentially identifiable images or data included in this article.

## Author contributions

JH: Writing – original draft, Writing – review & editing. WS: Writing – review & editing.
